# PSYCHOLOGICAL AND SOCIAL FACTORS ASSOCIATED WITH SLEEP HEALTH ACROSS ADULTHOOD

**DOI:** 10.1093/geroni/igz038.2862

**Published:** 2019-11-08

**Authors:** Soomi Lee, Orfeu M Buxton

**Affiliations:** 1 University of South Florida, Tampa, Florida, United States; 2 Pennsylvania State University, University Park, Pennsylvania, United States

## Abstract

Sleep is associated with all-cause mortality, cardiovascular disease, cognitive impairment, as well as daily social interactions and productivity. Studies often have focused on sleep duration only, lacking the ability to comprehensively understand the importance of age-related changes in varied facets of sleep health. Moreover, psychological and social factors that may be associated with sleep health in adulthood are still poorly understood. This symposium showcases contemporary endeavors towards understanding how diverse indicators of sleep health relate to psychological and social factors across adulthood. Paper 1 uses perceived job discrimination as a social stressor to test associations between perceived job discrimination and sleep health (difficulty falling/staying asleep, excessive daytime sleepiness, sleep duration) among working women. Paper 2 examines the relationship between personality traits and self-reported and actigraphy-measured sleep health (sleep duration, sleep quality, sleep latency, insomnia symptoms, wake-after-sleep-onset). Paper 3 uses daily diary data to examine the link between pain and sleep health (sleep disturbances, napping) in older adults’ everyday lives and test moderating effect of social support. Paper 4 examines sleep health (sleep latency, feeling unrested) as a mechanism linking physical activity and cognitive function. These papers use different project datasets that include diverse populations of middle-aged and older adults, such as the Sister Study, Midlife in the United States Study, and Daily Experiences and Well-being Study. At the end of these presentations, Dr. Buxton will discuss their theoretical and methodological contributions, and consider challenges and opportunities for future research.

